# The Basal Ganglia and Mesencephalic Locomotor Region Connectivity Matrix

**DOI:** 10.2174/1570159X21666230809112840

**Published:** 2023-08-09

**Authors:** Nicolás A. Morgenstern, Maria S. Esposito

**Affiliations:** 1 Champalimaud Research, Champalimaud Foundation, Lisbon, Portugal;; 2 Faculty of Medicine, University of Lisbon, Instituto De Medicina Molecular João Lobo Antunes, Lisbon, Portugal;; 3 Department of Medical Physics, Centro Atomico Bariloche, CNEA, CONICET, Av. Bustillo 9500, San Carlos de Bariloche, Rio Negro, Argentina

**Keywords:** Motor control, movement, action selection, pedunculopontine nucleus, cuneiform nucleus, striatum, substantia nigra

## Abstract

Although classically considered a relay station for basal ganglia (BG) output, the anatomy, connectivity, and function of the mesencephalic locomotor region (MLR) were redefined during the last two decades. In striking opposition to what was initially thought, MLR and BG are actually reciprocally and intimately interconnected. New viral-based, optogenetic, and mapping technologies revealed that cholinergic, glutamatergic, and GABAergic neurons coexist in this structure, which, in addition to extending descending projections, send long-range ascending fibers to the BG. These MLR projections to the BG convey motor and non-motor information to specific synaptic targets throughout different nuclei. Moreover, MLR efferent fibers originate from precise neuronal subpopulations located in particular MLR subregions, defining independent anatomo-functional subcircuits involved in particular aspects of animal behavior such as fast locomotion, explorative locomotion, posture, forelimb-related movements, speed, reinforcement, among others. In this review, we revised the literature produced during the last decade linking MLR and BG. We conclude that the classic framework considering the MLR as a homogeneous output structure passively receiving input from the BG needs to be revisited. We propose instead that the multiple subcircuits embedded in this region should be taken as independent entities that convey relevant and specific ascending information to the BG and, thus, actively participate in the execution and tuning of behavior.

## 1 INTRODUCTION

The mesencephalic locomotor region (MLR) was first described in the 1970s for its ability to induce coordinated locomotion upon electrical microstimulation in decerebrate and intact cats [1, 2]. After the initial MLR discovery, several groups have demonstrated its existence in all vertebrates studied to date, from lampreys to humans [3-9]. The initial functional description of a midbrain locomotor center was then followed by the dissection of anatomical regions encompassing the MLR: the cuneiform nucleus (CnF), the pedunculopontine nucleus (PPN) [10], and its adjacent medial extension [5, 11, 12]. Within these regions, a wide variety of different cell types coexist, which can be stratified by neurotransmitter phenotype, gene expression, axonal projection, and topographical organization. This cellular heterogeneity is paralleled by a diversity of functional roles that, over the years, have expanded far beyond the original description of a midbrain locomotor center. MLR has been implicated in many functions, including escape responses, atonia, freezing, rapid eye movement (REM) sleep, wakefulness, arousal, reward, and autonomic functions, raising the question of how such a diversity of functions can coexist within such a small region [13-15]. However, traditional techniques based on low-threshold electrical microstimulation or local neurochemical microinjection lack the cellular resolution required to distinguish the individual functional role of each neuronal subpopulation residing intermingled within the MLR. In recent years, the complexity of the MLR, as revealed by anatomical organization and function, has begun to be disentangled by incorporating tools allowing the targeting of specific circuit elements with exquisite cellular resolution. By applying cell-type specific viral tracing tools, optogenetics, and neuronal activity recording in behaving animals, it was possible to uncover the high degree of input-output specificity and functional selectivity of individual MLR components, which we will describe in detail in the first section of this review.

The classical model of motor system function proposes that movement is elicited by the disinhibition of basal ganglia (BG) output targets [16]. Consequently, a lot of attention has been placed on the BG to MLR input. In this framework, locomotion is proposed to be elicited by a reduction of MLR inhibition exerted by substantia nigra reticulate (SNr), the main BG output in rodents. However, modern techniques have now made it possible to demonstrate not only that several BG nuclei send preferential inputs to specific subsets of MLR neurons [8, 17-19] but also that specific MLR neuronal subpopulations convey selective feedback to precise BG nuclei, which markedly impact BG function [19-25]. The strong interconnectivity between these two structures may underlie their shared role in motor, sensory and cognitive functions. The MLR - BG interplay will be the topic of the second section of this review.

A significant consequence of the unveiling cellular heterogeneity of MLR and BG neuronal composition is the increasing methodological complexity required to decipher their reciprocal connectivity. An emerging organizational principle of BG posits that specific ensembles of neurons are interconnected, creating parallel subcircuits controlling particular aspects of behavior [26, 27]. We propose that this principle extends to the MLR organization that can now be uncovered by novel intersectional tools allowing the discrimination of intermingled neuronal subpopulations by, for example, neurotransmitter phenotype and axonal targets. Therefore, in this work, we will review recent studies that have taken advantage of novel cell-type-specific tools to address the connectivity and functional relevance of precise MLR-BG pathways. In doing so, we will discuss novel findings that challenge our understanding of MLR function as a mere BG output region. Moreover, we will highlight the concept that the intrinsic cellular diversity underpinning MLR and BG circuits requires a thorough dissection of the precise neuronal populations and pathways engaged in particular behaviors. Deciphering the precise connectivity matrix between MLR and BG circuit elements is the foundation for understanding the computational process underlying the selection and execution of a desired motor command.

## 2 INTRINSIC MLR ORGANIZATION AND FUNCTION

Despite the limited anatomical resolution of traditional techniques based on low-threshold electrical microstimulation or neurochemical microinjections, early studies converged on two major nearby regions possibly involved in eliciting a locomotor response: CnF and PPN, defined by the presence of cholinergic neurons [1, 2, 5, 10, 28]. Furthermore, although often overlooked, the effective locomotion-inducing PPN site was reported to extend more medially than cholinergic neurons to the adjacent mesencephalic reticular formation (mRT), sometimes referred to as the midbrain extrapyramidal region [10, 11, 29-33]. However, due to the technical limitations of the traditional strategies implemented, there was a long debate on the precise functional role of CnF *versus* PPN/mRT MLR subregions in the control of gait and posture. While some studies pointed to CnF as the nucleus responsible for initiating locomotion and gait control [1], others found similar results in PPN [34]. Even more, other studies found a reduction in muscle tone when PPN was activated, which was related to a role in muscle atonia associated with REM sleep [14]. These discrepancies have recently been resolved by the implementation of novel tools allowing us to visualize and modulate brain regions with great spatial precision [35].

Traditional techniques also lack the ability to distinguish the functional role of intermingled diversity of neuronal types. At least three neuronal subtypes have been described to coexist within the MLR: glutamatergic [36] and GABAergic neurons [37] are distributed in PPN, CnF, and mRT, and cholinergic neurons (Ch5 group) that are located specifically within the PPN [38-40]. This neuronal heterogeneity was also shown in the human MLR [41]. Nowadays, technological advances based on cell-type specific targeting make it possible to address the functional role of particular circuit elements with unprecedented resolution [35]. The combination of selective activation/silencing, input/output mapping and assessment of endogenous activity of specific neuronal subpopulations has recently started to unveil the complexity of MLR organization and function.

These modern approaches were first applied to uncover which MLR neuronal subtype was responsible for eliciting full-body locomotion without distinction between MLR subregions. Selective optogenetic stimulation of individual MLR neuronal subtypes demonstrated that activation of glutamatergic MLR neurons is sufficient to drive locomotion from rest [8, 42, 43]. In contrast, their optogenetic silencing in head-fixed mice running on a spherical treadmill reduces locomotion speed and eventually induces full stopping [8]. In line with these results, *in vivo* electrophysiological recordings of light-identified glutamatergic MLR neurons during spontaneous locomotion evidenced the existence of two neuronal subpopulations: one whose activity correlates with the locomotor state (running *vs*. rest) and another one that correlates with locomotor speed [8].

Regarding the functional role of cholinergic MLR neurons, evidence from several labs agrees that their photo-stimulation is not able to evoke locomotion from rest, but it elicits a modulatory effect on ongoing locomotion ([8, 17, 21, 44], see section 2.2 below).

Finally, optogenetic activation of GABAergic MLR neurons reduces locomotion speed putatively by local inhibition of non-GABAergic surrounding neurons while failing to induce movement from a resting state [8, 45]. Taken together, these findings provide strong evidence for the role of glutamatergic MLR neurons in promoting locomotion.

The abovementioned work was followed by several studies aiming to tease out the contribution of specific elements of the MLR to the control of gait, posture, learning, and sleep-wake cycle, highlighting an unexpected complexity of this region. In the following sections, we will summarize and discuss these findings with a focus on cellular diversity and circuit specificity. We will first describe the role of glutamatergic, cholinergic, and GABAergic MLR neurons in the control and tuning of locomotion, highlighting the differential contribution of CnF, PPN, and mRT MLR subregions (sections 2.1 to 2.3). We will continue by outlining other functional roles that have been linked to specific PPN neuronal subtypes (section 2.4). Finally, we will delineate the intrinsic MLR connectivity in an attempt to understand how its cellular and functional diversity might be integrated to achieve an adaptive behavior (section 2.5).

### 2.1 Diversity of Glutamatergic MLR Neurons

#### 2.1.1 Glutamatergic CnF Neurons

**Fig. (1):**
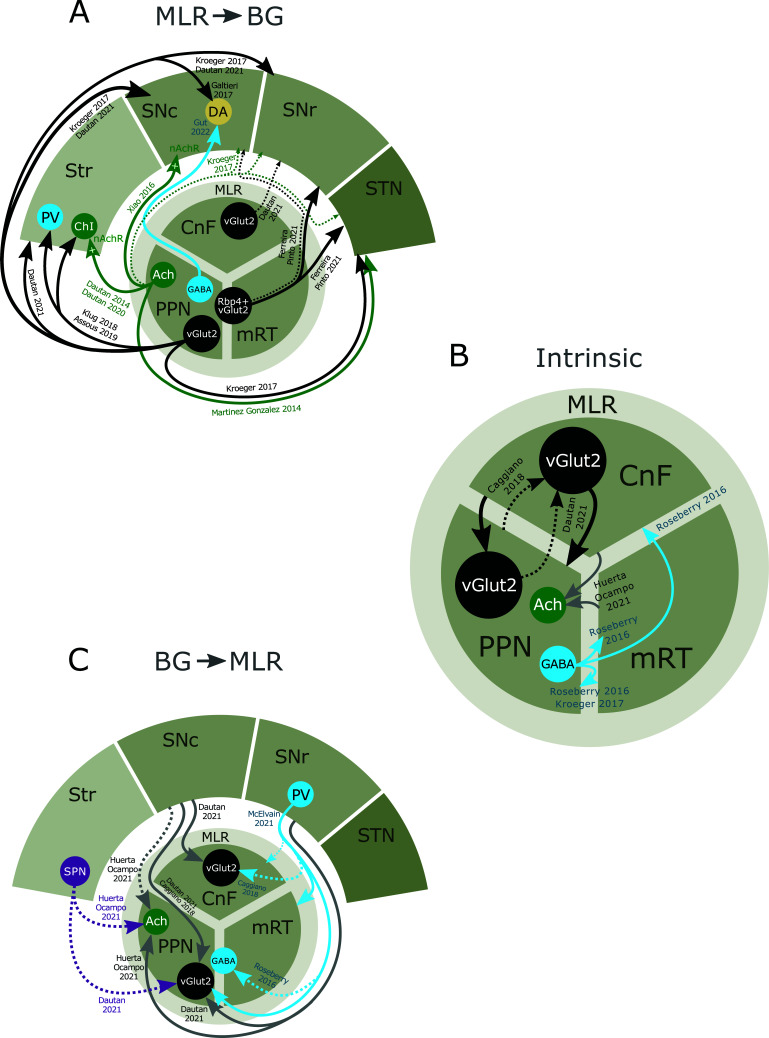
MLR intra- and inter-connectivity with the BG. (**A**) MLR projections to the main BG nuclei divided by their subregion of origin (PPN/mRT and CnF) and neuronal subtype (vGlut2, black; Ach, cholinergic neurons, green; GABA, GABAergic neurons, light blue). When reported in the literature, specific neuronal targets are also depicted (PV, parvalbumin interneurons; ChI, cholinergic interneurons; DA, dopamine neurons). The reference describing each specific connection is included nearby the arrow representing it. Dotted lines represent minor connections. For simplicity, only MLR neuronal types with known connectivity were included. (**B**) Connectivity within the MLR subregions. Neuronal type of origin/target are included when described in the literature. Gray arrows represent connections where the neuronal subtype originating from is not known. (**C**) BG projections to the MLR divided by their nuclei of origin (SPN, spiny projection neuron).

Once glutamatergic neurons were identified as the neuronal substrate responsible for triggering MLR-induced locomotion, the question arose whether there are functional differences between neurons located in different subregions within the MLR. Recent efforts have led to converging evidence for the role of glutamatergic CnF neurons in high-speed locomotion, such as escape responses. In support, optogenetic stimulation of glutamatergic CnF neurons induced reliable full-body locomotion from rest [17, 19, 44]. By increasing the stimulation frequency of glutamatergic CnF neurons, animals increased their locomotion speed [17, 19, 44] and transitioned from low-speed alternating walk and trot to higher-speed synchronous gaits such as gallop and bound [17, 44]. Photo-activation of glutamatergic CnF neurons increased the motor drive of bilateral flexor and extensor muscles, impacting positively on the locomotor rhythm [19, 44]. Pharmacogenetic inhibition of glutamatergic CnF neurons reduced the speed of ongoing locomotion on a treadmill [17, 44] and the escape response elicited by an air puff. In contrast, low-speed exploration was not affected [17]. Interestingly, although locomotor speed is scaled by light intensity during optogenetic stimulation of CnF neurons, animals were still able to brake and turn to avoid obstacles, demonstrating that the evoked response can be integrated with sensory inputs to elicit an adaptive motor output [46]. Anatomically, glutamatergic CnF neurons receive little input from BG nuclei but strong afferences from midbrain structures (Fig. **1C**) [17, 19]. In addition, the efference connectivity of glutamatergic CnF neurons is directed to the midbrain and medullary structures [17, 19], exhibiting only minor projections to BG areas restricted to substantia nigra reticulata (SNr) (Fig. **1A**) [19]. Overall, known connectivity patterns and functional data support the role of glutamatergic CnF neurons in the regulation of high-speed locomotion.

#### 2.1.2 Glutamatergic PPN Neurons

In contrast to glutamatergic CnF neurons, photo-stimulation targeted to glutamatergic PPN neurons yielded divergent results between different studies. Notably, optogenetic activation of glutamatergic PPN neurons was described to either trigger locomotion [17] or to have no effect on resting mice [19, 44], while causing ongoing locomotion to speed-up [47] or decelerate and eventually stop [19, 44, 47]. Nevertheless, locomotion elicited by glutamatergic PPN photo-activation was less reliable than photo-activation of glutamatergic CnF neurons, and also had longer onset latency and required higher stimulation frequencies (20-50Hz) [17]. High-frequency glutamatergic PPN stimulation only elicited low-speed responses limiting locomotion to walk and trot gaits, highlighting the difference from CnF-evoked locomotion [17]. It is important to note that Caggiano and collaborators were the only ones to apply such a high-frequency photo-stimulation protocol which may explain, at least partially, the observed discrepancies [19].

Moreover, prolonged chemogenetic activation of glutamatergic PPN neurons increased the distance traveled in an open field, supporting a positive role for these neurons in locomotion [48]. When low-frequency stimulation was used to activate glutamatergic PPN neurons in a mouse walking on a grid, an increase in slips was found, suggesting a role of PPN in sensory-motor integration [19]. Strikingly, photo-activation of glutamatergic PPN neurons varies its impact on muscle activity depending on the animal behavioral state (walking *versus* rest), stimulus duration, and the phase of the step cycle targeted, suggestive of a descending information control by spinal locomotor circuits [44]. Overall, unilateral glutamatergic PPN stimulation at rest evoked excitatory motor responses in flexor rather than extensor muscles bilaterally which could indicate postural adjustments preceding locomotion [19, 44] while during ongoing locomotion it induced a reduction in the walking speed [44].

The abovementioned results were based on gain-of-function experiments that may elicit a behavioral effect by perturbing downstream circuits [49]. Complementary pharmacogenetic or optogenetic silencing experiments of glutamatergic PPN neurons showed a reduction in the speed of ongoing locomotion on a treadmill [17, 44] and in the exploratory behavior in a hole-board test [17]. However, optogenetic silencing of glutamatergic PPN neurons did not show significant effects in the open field nor in the grid test [19]. These controversial findings might originate from the long-known heterogeneity of the PPN structure. Regardless of the experimental approach chosen to target them, be it neuronal activity [8, 17, 25, 50-52], biophysical properties [19], neurochemical composition [53], or anatomical tracing [50], PPN neurons were commonly characterized by a remarkable degree of variability which is in stark contrast to glutamatergic CnF neurons [19] and may underlie the diversity of behaviors attributed to PPN (section 2.4 below).

#### 2.1.3 Ascending and Descending Subpopulations of Glutamatergic PPN/mRT Neurons

Anatomically, glutamatergic PPN neurons receive strong input from several BG nuclei and display a brain-wide output connectivity matrix (Fig. **1**) [17, 19]. A recent study has begun to unravel the heterogeneity of glutamatergic PPN neurons by stratifying neuronal subpopulations based on their selective axonal projection pattern [25]. In this work, two intermingled glutamatergic neuronal subsets spanning PPN and mRT (PPN/mRT) were identified: a descending subpopulation that projects to the spinal cord and is recruited during rearing and, to a lesser extent, during locomotion; and an ascending subpopulation which targets the SN, the entopeduncular (EP) and the subthalamic nucleus (STN) and is engaged during forelimb movements such as grooming and handling [25]. SN projecting glutamatergic PPN/mRT neurons were identified by the expression of Cre in Rbp4-Cre mice. Moreover, MLR neurons innervating the medullary reticular formation are located in the CnF and PPN/mRT subregions and do not overlap with ascending SN projecting MLR neurons. These data demonstrate the presence of at least three separate populations of glutamatergic MLR neurons based on their axonal targets: descending medullary projecting neurons, descending spinally projecting neurons, and ascending SN projecting neurons [25].

Perturbation experiments further enhanced our understanding of the function of glutamatergic PPN/mRT neurons. Optogenetic activation of spinally projecting glutamatergic PPN/mRT neurons evoked body stretching, whereas photo-inhibition during rearing episodes reduced body extension [25]. Moreover, optogenetic stimulation of this neuronal subpopulation failed to induce locomotion from rest but increased stepping probability in conditions that favor locomotion [25]. Taken together, these results demonstrate that spinally projecting glutamatergic PPN/mRT neurons control postural body adjustments involved in rearing and locomotion. In contrast, optogenetic modulation of ascending glutamatergic PPN/mRT neurons elicits a complex behavioral response associated with its multiple BG targets (see section 3.2.1 below). Briefly, photo-activation of ascending glutamatergic PPN/mRT neurons evoked the stopping of ongoing movement [25]. Based on these findings, it is conceivable that the inconsistent results on glutamatergic PPN function described above [17, 19, 44, 47] may have arisen from differences in injection sites and fiber-optic placements targeting more the ascending or the descending glutamatergic PPN neuronal subsets, leading to either stopping or walking, respectively. Overall, given the weak contribution of spinally projecting PPN/mRT neurons to the onset of locomotion, the ability of MLR to trigger a locomotor response is most likely achieved through its downstream connectivity to medullary targets.

### 2.2 Cholinergic PPN Neurons

Several studies have recently addressed the role of cholinergic PPN neurons in the control of locomotion. All of them have consistently shown the inability to initiate locomotion by optogenetic activation of this neuronal subtype [8, 17, 44]. However, the impact of cholinergic PPN neurons on ongoing locomotion remains a matter of debate. While some authors have found a positive modulation of the animal's ongoing running speed upon cholinergic photo-activation [8, 21], others have shown that optogenetic activation of cholinergic cells slows down and even stops ongoing locomotion [17, 44]. Moreover, the only study assessing the effect of photo-inhibition of cholinergic PPN neurons found a reduction in ongoing locomotion speed [21]. To better understand the circuit mechanism underlying the evoked motor output, Josset and colleagues performed simultaneous EMG recordings and kinematic analysis of hindlimb muscles during optogenetic stimulation in standing and running mice. Short-pulse stimulation of cholinergic PPN neurons elicited long-latency responses and strong bilateral activation of extensor muscles at the expense of flexor muscles at rest [44]. During locomotion, a similar stimulation protocol evoked a prolongation of the stance phase resulting in a slowing of the locomotor rhythm [44]. These conflicting results could originate from differences in stimulation patterns which may differentially impact downstream circuits at various time-scales. Moreover, it could also involve state-dependent control of input signals by local circuits as observed during different locomotor phases [44]. Finally, it is also possible that intermingled cholinergic subpopulations with different functional roles may contribute to the inconsistent results [21] (as discussed in section 3.3.1), resembling the observations from glutamatergic PPN neurons. Interestingly, cholinergic PPN neurons are characterized by an extensive brain-wide efferent connectome [54, 55] (Fig. **1**). However, single-neuron tracing experiments demonstrated that an individual cholinergic PPN neuron could innervate the most known output regions [56]. The use of techniques capable of identifying neuronal subpopulations based on neurotransmitter phenotype and axonal targets will make it possible to demonstrate at a population level whether segregated neuronal subsets with specialized projection patterns coexist within the PPN.

### 2.3 GABAergic MLR Neurons

There is a limited number of studies addressing the connectivity map and functional role of GABAergic MLR neurons. Within PPN, it has been found that GABAergic neurons display a heterogeneous rostrocaudal distribution: rostral PPN contains higher densities of inhibitory neurons that decrease progressively towards posterior levels, except for a concentrated group of GABAergic neurons in the most caudal part of PPN [57]. In terms of outputs, GABAergic PPN neurons send both ascending and descending projections [45]. Prominent ascending targets are dopaminergic lateral SN compacta (SNc) neurons and, to a lesser extent, ventral tegmental area (VTA), extended amygdala, hypothalamus, and thalamus [45]. Moreover, they also display significant projections to the dorsal raphe, superior colliculus, and hindbrain [45]. These results contrast with previous work that failed to observe GABAergic efferent fibers extending beyond the injection site [22]. Incoming inputs to GABAergic MLR neurons have been assessed by monosynaptic rabies tracing, revealing sparse innervation from BG, originating mainly from SNr, but major afferents from the superior colliculus, dorsal raphe, bed nucleus of the stria terminalis (BNST), and laterodorsal tegmentum (Fig. **1**) [8].

The role of GABAergic MLR neurons in movement control has begun to be unraveled by perturbation experiments and recordings of neuronal activity *in vivo*. Optogenetic stimulation of GABAergic MLR neurons elicited a reduction of ongoing locomotion [8], which could be explained by the local inhibition of non-GABAergic MLR neurons [8, 22]. When the endogenous activity of photo-identified GABAergic MLR neurons was assessed in behaving mice, a variety of responses were observed, with neurons firing during rest periods and others during running episodes [8]. In contrast, another study showed that prolonged pharmacogenetic activation of GABAergic PPN neurons induced a long latency increase in the total distance traveled characterized by low-speed locomotor episodes interrupted by stop bouts [48]. The circuit mechanism underlying this unexpected finding is not yet well explained but may involve a balance between the intrinsic MLR connectivity and long-range projections to BG. Notably, another recent study uncovers the functional role of GABAergic PPN input to SNc in the control of purposive behaviors (see section 3.3.1) [45]. Taken together, these findings highlight that the well-described functional heterogeneity of MLR extends beyond glutamatergic and cholinergic PPN neurons, also reaching GABAergic MLR neurons.

### 2.4 Non-motor Functional Roles of PPN

The PPN region has long been linked to the ascending reticular activating system (ARAS), capable of regulating cortical arousal and promoting wake, but also to the control of REM sleep [58-61]. These controversial roles could again be explained by the heterogeneity of MLR neuronal composition. However, even with the advent of new tools allowing to dissect the contribution of selective neuronal subtypes to specific brain states, the findings from different laboratories remain inconsistent. Pharmacogenetic activation of cholinergic PPN neurons during the sleep phase did not alter the total amount of sleep or wake state, but it induces a light non-REM sleep characterized by a reduction in EEG slow waves [22]. In contrast, optogenetic activation of cholinergic PPN neurons induces REM sleep [62]. *In vivo* recordings of cholinergic PPN neurons showed that they are maximally active during wake and REM sleep state [51]. However, cholinergic PPN neurons are also active during slow oscillations induced by general anesthesia, a state that resembles slow-wave non-REM sleep [56]. In this experimental setup, the administration of a broad cholinergic agonist into the PPN induced an increase in cortical gamma activity during slow oscillation, suggesting a contribution of midbrain structures to slow-wave sleep [56].

Glutamatergic and GABAergic PPN neurons have also been linked to the control of the sleep-wake cycle. Pharmacogenetic activation of glutamatergic PPN neurons during the sleep phase induces quiet wakefulness, while pharmacogenetic silencing elicits a modest increase in sleep [22]. In contrast, pharmacogenetic activation of GABAergic PPN neurons induced a mild reduction of REM sleep [22]. The firing profile of glutamatergic PPN neurons during the sleep-wake cycle allowed the identification of three neuronal subsets: neurons maximally active during wake and REM sleep, maximally active only during REM, or only during wake [51]. Moreover, different discharge profiles from identified GABAergic PPN neurons were observed during the sleep-wake cycle, with neurons maximally active during wakefulness and REM sleep and others selectively recruited during REM sleep [51]. These data underscore the functional heterogeneity existing in PPN, even within neurotransmitter-identified neurons.

Individual components of MLR subcircuits have also been linked to the limbic system suggesting a strong modulation of locomotion by higher-order states, such as emotions [15, 63]. Glutamatergic PPN neurons receive direct input from the central amygdala and BNST [8, 19], and GABAergic MLR neurons are contacted by BNST [8]. Moreover, reciprocal connections between MLR and periaqueductal gray have been described [17]. Although a thorough description of this interconnectivity is beyond the scope of this review, it is important to highlight that the MLR is embedded in a brain-wide network that extends beyond the classically described motor system and includes, for example, defensive circuits underlying the strong impact of emotions on locomotion.

Beyond brain states, earlier studies demonstrated that the PPN is required for reinforcement learning. PPN-lesioned rats display impairments in learning a radial maze task [64] and a lever-press operant task [65, 66]. Moreover, the inactivation of posterior PPN prevents shifts in behavior associated with changes in action-outcome contingency [67]. These results support the idea that PPN neurons participate in learning action-outcome associations.

PPN neurons have also been found to respond to rewards. Recordings of PPN neurons in monkeys have shown an increase in firing upon reward or cues that predict reward that scales with reward magnitude [68, 69]. Later on, Norton and colleagues demonstrated that different subsets of PPN neurons encode reward and movement in rats performing a spatial navigation task [70]. However, another study performed in mice trained in an odor-cued spatial choice task showed overlapping PPN neuronal populations encoding movement direction and reward outcome [71]. Interestingly, the activity of unidentified PPN neurons reflects the immediate prior experience representing not only past outcomes (rewarded or non-rewarded) but also previous selected actions, correlating with and predicting the upcoming choice [72]. These data support the intriguing contribution of PPN to action selection.

The aforementioned findings did not discriminate between the different neuronal types coexisting in PPN. However, the spiking properties of recorded PPN neurons suggested that reward-responsive neurons belong to a cholinergic origin [70]. Indeed, selective photo-excitation of cholinergic PPN neurons reverses the aversion for a light compartment in a place conditioning task, whereas photo-inhibition of these neurons reduces the preference for a dark compartment, indicating a role in reward processing [21]. Notably, the impact of cholinergic PPN neurons on the reward system is specifically linked to VTA-projecting neurons and not to ventral SNc-projecting ones (see section 3.3.1).

Interestingly, it was recently described that the population activity of cholinergic PPN neurons not only responds to reward but also signals error trials during reversal learning, suggesting that they may encode changes in reward contingency [73]. In line with their endogenous activity, pharmacogenetic silencing of cholinergic PPN neurons suppresses reversal learning and increases perseverative errors, while activity enhancement improves reversal learning [73].

Notably, PPN/mRT neurons were recently shown to become specifically activated by an auditory go cue in a delayed-response task in which mice learn to lick left or right for a reward [74]. PPN/mRT neuronal activity did not reflect a mere auditory response but rather a learned response to a sound associated with the go cue [74]. Moreover, neuronal activity triggered by the go cue in PPN/mRT not only displayed a short-latency but even preceded the auditory cue response in the motor cortex [74]. Furthermore, the authors demonstrated that glutamatergic PPN/mRT neurons convey this sensory information to the motor cortex. Photo-stimulation of thalamic PPN/mRT axonal terminals replacing the go cue evoked a licking response in the correct direction and a similar cortical response to the one elicited by the actual auditory signal [74]. Moreover, photo-inhibition of glutamatergic PPN/mRT neurons projecting to the thalamus during the go cue blocked go-cue-triggered movement. Altogether, this work demonstrated that PPN/mRT conveys learned sensory information to the motor cortex *via* the thalamus to release the planned movement [74].

In summary, different MLR neurons have been found to encode sensory stimuli, reward, reward contingency, ongoing movements, preceding actions, and behavioral states (wake or sleep). Altogether, these results underscore the notion that to disentangle the endogenous role of PPN neurons, it may not suffice to target specific neuronal subtypes based on neurotransmitter phenotype. Instead, it may require a combination of approaches manipulating and recording the activity of selective neuronal subpopulations targeted by neurotransmitter identity, gene expression profile, location, and/or projection pattern applied to multiple behavioral contexts and paradigms. Moreover, based on the intricate interplay between MLR and BG nuclei, it is conceivable that at least some of the information encoded by MLR neurons is not only driven by BG output but also fed back from MLR to BG circuits contributing in this way to its known role in action selection and reinforcement learning.

### 2.5 MLR Intrinsic Connectivity

The functionally described MLR was historically defined to lie within three subregions: the CnF, the PPN, and the adjacent mRT. However, recent findings challenge the view of a unified MLR by demonstrating distinct functional roles for each specific subregion in movement control. Such functional differences are supported by dedicated neuronal subcircuits embedded within the midbrain locomotor area. To better understand whether MLR subregions work in conjunction to control behavior, it becomes important to investigate whether and how each circuit element is interconnected with one another. Unfortunately, there are only a handful of studies vaguely addressing this issue. For example, individual cholinergic PPN neurons have been found to give rise to a wide network of local axon collaterals and synapses before extending their long-range projecting axons to more distant targets [56]. However, which specific neuronal PPN subpopulations are targeted by cholinergic PPN neurons was not assessed [56]. Notably, these results suggest a strong modulation of cholinergic PPN neurons on the activity of neighboring cells. In turn, rabies tracing experiments from cholinergic PPN neurons identified direct inputs from CnF and mRT neurons [18] (Fig. **1B**). Although the neurotransmitter phenotype of the identified input neurons was not addressed, these findings support the possibility of a direct impact of putative locomotor signals on cholinergic PPN activity.

Furthermore, work addressing the local connectivity of glutamatergic neurons found reciprocal connections between CnF and PPN, although there were some inconsistencies in circuit details between different studies [17, 19] (Fig. **1B**). While Caggiano and colleagues found that CnF input to glutamatergic PPN neurons dominates over PPN input to glutamatergic CnF subpopulation [17], Dautan *et al.* showed no CnF input to glutamatergic PPN neurons but strong PPN input to glutamatergic CnF neurons [19] (Fig. **1B**). It is important to note that the monosynaptic rabies tracing strategy used does not allow to identify the neurotransmitter phenotype of the input neurons. In terms of output, it was demonstrated that glutamatergic CnF neurons project to PPN and that glutamatergic PPN neuron send a weaker innervation to CnF [19] (Fig. **1B**). Although possible, whether glutamatergic CnF and PPN neurons have reciprocal connectivity remains to be determined.

Much less is known regarding the intrinsic connectivity of GABAergic MLR neurons. It was first described that PPN GABAergic axonal projections terminate within PPN, which may indicate that they function as local interneurons [22, 37] (Fig. **1B**). Furthermore, it was shown that GABAergic MLR boutons surround the cell-bodies and proximal dendrites of cholinergic PPN neurons [37]. In line with this finding, MLR photo-stimulation of GABAergic MLR neurons inhibited non-GABAergic neurons *in vitro* and *in vivo* [8] (Fig. **1B**), suggesting a possible role in sculpting MLR dynamics.

The data obtained so far reveal an intricate connectivity matrix within MLR with potential feedforward and feedback loops and neuromodulation as potential circuit mechanisms underlying the computation of MLR-dependent motor outputs. Deciphering the precise intrinsic connectivity of specific MLR circuit components is essential to understand whether the MLR works in concert to generate an adaptive behavioral output or, conversely, whether there are parallel subcircuits that independently control specific aspects of the selected motor program. In the first scenario, the use of the term MLR is founded on the basis of a common functional output. However, in the second scenario, the term MLR undermines the sense of independent parallel processes running through different circuit channels, in which case it would be more appropriate to refer precisely to the subcircuit under study.

## 3 INTERPLAY BETWEEN BG AND MLR

The BG are a series of interconnected subcortical nuclei involved in learning, selecting, and executing actions [27, 75-77]. Most connections within the BG are inhibitory and mediated by GABA. The striatum is the first and largest input nucleus of the BG and receives its major afferents from the cortex and the thalamus [75, 78]. The spiny projection neurons (SPNs) are the most abundant and the only neuronal type projecting outside the striatum [76, 79]. SPNs can be divided into two populations based on their postsynaptic long-range targets and the expression of mutually exclusive dopamine receptor subtypes [79]. The first population expresses dopamine-1-receptor (D1R) and sends projections to the EP and SNr, the major BG output nucleus. Thus, the striatum directly modulates SNr activity through monosynaptic inhibition (striatonigral or direct pathway). The second population of SPNs expresses dopamine-2-receptor (D2R) and projects to the globus pallidus externus (GPe). In this manner, the striatum indirectly disinhibits SNr output by inhibiting GPe neurons that inhibit glutamatergic STN neurons projecting to the EP and the SNr (striatopallidal or indirect pathway). SNr neurons project outside the BG circuit (see below), modulating brainstem, midbrain, and thalamocortical activity, and closing the cortico-BG-thalamic loop [78, 80]. Moreover, the topographic organization originating in the striatum is maintained throughout other BG elements, which could be interpreted as parallel subcircuits controlling specific behaviors [26]. Signals from specific BG subcircuits are then outputted as parallel channels of information to distant brainstem structures [26, 80]. Moreover, the output information is also fed back *via* thalamo-cortical pathways to corresponding BG subcircuits, forming topographically organized long-range closed loops [26]. Importantly, SNc fibers reach several BG nuclei, exerting dopamine-mediated neuromodulation at multiple points of the circuit [76, 81].

The MLR and BG display reciprocal interconnectivity. However, some MLR regions preferentially project to specific BG centers, avoiding others. Similarly, BG nuclei do not innervate MLR structures homogeneously, with some connections predominating over others. In this section, we will review the ascending MLR inputs to the major BG structures (Striatum, SNr, SNc, *etc*.) and describe the innervation that MLR receives from these diverse nuclei. When possible, we will distinguish the specific neuronal types (glutamatergic, cholinergic, GABAergic) and/or the specific MLR substructures (PPN/mRT and CnF) involved. Finally, we will discuss how an accurate characterization of input-output connectivity maps impacts our understanding of circuit function and motor control processing.

### 3.1 MLR Connectivity with the Striatum

The striatum is the largest nucleus of the BG and the main recipient of inputs from the cortex and thalamus [75]. This nucleus is mostly composed of SPNs, representing more than 95% of the neurons in this structure [79, 82]. The remaining 5% of the neurons in the striatum are predominantly GABAergic interneurons of different subclasses (fast-spiking (FSI), tyrosine hydroxylase (TH), low-threshold spike (LTS) interneurons, *etc*.) and a small and homogeneous population of cholinergic interneurons (ChIs) [83]. Cortical input to the striatum is topographically organized, defining different anatomical regions with particular functions based on their afferent connectivity. Using traditional approaches, three main striatal regions were defined, the dorsolateral striatum (DLS), the dorsomedial striatum (DMS), and the ventral striatum [84]. These areas are thought to be related to sensorimotor, associative, and limbic functions, respectively [84]. However, recent studies based on new methods allowed refining this classification with increased granularity, unraveling almost 30 different striatal anatomo-functional domains [85].

#### 3.1.1 Inputs from PPN Neurons to Striatum

Our current knowledge of the MLR inputs to the striatum is highly restricted to those originating in the PPN. In recent years, there have been advances in our understanding of both glutamatergic and cholinergic innervation of this structure by PPN neurons while leaving GABAergic projections underexplored. Given the potential impact of direct PPN control of the main BG input structure on circuit processing and action planning, in this section, we review recent findings deciphering the cellular specificity and functional relevance of the PPN-to-striatum circuit.

Viral-mediated fluorescent labeling of glutamatergic neurons (vGlut2) from PPN and CnF neurons revealed that only PPN fibers reach the striatum [19] (Fig. **1A**). However, this experimental approach fails to inform axons’ specific neuronal targets. In that sense, circuit mapping experiments in brain slices showed that glutamatergic PPN neurons preferentially project to ChIs and FSIs and, to a lesser extent, also to TH and LTS interneurons while avoiding SPNs [23] (Fig. **1A**). The aforementioned selective ascending innervation of interneurons was found ipsi- and contralaterally in the dorsal anterior striatum. Functionally, the activation of these excitatory axons *ex vivo* results in feed-forward inhibition of SPNs [23]. *In vivo*, the same activation induces a short-latency increase in spike frequency of FSIs and ChIs, followed by a late-onset decrease in SPNs firing rate [23]. When recruited unilaterally in awake mice, the activation of these fibers produces ipsiversive head rotations, consistent with the inhibition of striatal output by feed-forward inhibition of striatonigral and striatopallidal pathway neurons [23]. Consistently, a previous study showed that striatal ChIs preferentially receive non-cholinergic excitatory inputs from the PPN (Fig. **1A**). These inputs are located in the ChIs’ somata and proximal dendrites [86], indicating that their activity is, at least in part, controlled by putative glutamatergic PPN fibers. Besides the direct feed-forward inhibition of SPNs exerted by the activation of ChIs by PPN glutamatergic axons, acetylcholine released from ChIs could also impact other neurotransmitters systems since it could presynaptically affect the release of glutamate [87, 88] and/or dopamine [89], complicating the prediction of the net effect of PPN activity on striatal microcircuitry. Together, these findings suggest that PPN ascending glutamatergic inputs to the striatum, rather than directly contacting the abundant SPNs, preferentially synapse onto striatal interneurons that indirectly modulate SPNs activity.

Taking into consideration the existence of non-overlapping independent ascending and descending populations of projecting glutamatergic PPN neurons [25], we could, in principle, assume the contribution of the former to the modulation of striatal function. An open question is whether these PPN axons reaching the striatum are also originating in the SN-projecting Rbp4+ population of glutamatergic neurons (see below and [25]) or they arise from a dedicated neuronal subpopulation. If they are collaterals from that population, that would imply that when these neurons are recruited during the execution of forelimb behaviors, SPNs are strongly inhibited by feed-forward inhibition conveyed by both FSIs and ChIs [23]. In this manner, direct control of interneurons by sparse PPN axons could elicit strong behavioral effects since interneurons target multiple SPNs and may shut down any striatal-dependent motor program being executed. If instead, the PPN glutamatergic axons projecting to the striatum originated in an independent ascending population of neurons, then their control over SPNs would not be directly linked to PPN/mRT-dependent behaviors.

PPN cholinergic neurons innervate the striatum following a topographical distribution that avoids the striosomes [20]: rostral PPN cholinergic inputs target the anterior DLS matrix while caudal PPN cholinergic inputs innervate the nucleus accumbens (NAc) and the dorsal striatum matrix [20]. Regarding their specific neuronal targets, PPN cholinergic neurons preferentially synapse onto striatal ChIs when compared to D1R- or D2R-SPNs [24] (Fig. **1A**). This innervation is excitatory in nature, likely due to the activation of ChIs’ nicotinic receptors by acetylcholine [24] (Fig. **1A**). *In vivo*, the excitation of ChIs by PPN cholinergic fiber activation results in indirect SPNs inhibition. On the other hand, parvalbumin (PV) neuron activity *in vivo* is barely affected by PPN cholinergic fiber activation, indicating little or no cholinergic PPN synapses in these inhibitory interneurons [24]. In behaving rats, silencing the PPN cholinergic input to the DLS impairs their ability to form habitual behaviors [24], evidencing their role in normal striatal function. Thus, similarly to glutamatergic connectivity to the striatum, cholinergic innervation from PPN is also highly selective to interneurons but in this case, further limited to ChIs.

#### 3.1.2 Inputs from Striatum to PPN

In opposition to the PPN to striatum connectivity, our knowledge of direct striatum to MLR connectivity is scarce, with a handful of studies showing only a moderate innervation from SPNs to vGlut2+ PPN and cholinergic PPN neurons [17-19] and a negligible input to glutamatergic CnF neurons [17, 19] (Fig. **1C**). Thus, although these studies described direct striatal inputs from SPNs to PPN neurons (Fig. **1C**), they seem to represent a relatively small proportion of BG inputs to MLR, and their functional relevance has not been addressed so far.

### 3.2 MLR Connectivity with the SNr

**Fig. (2):**
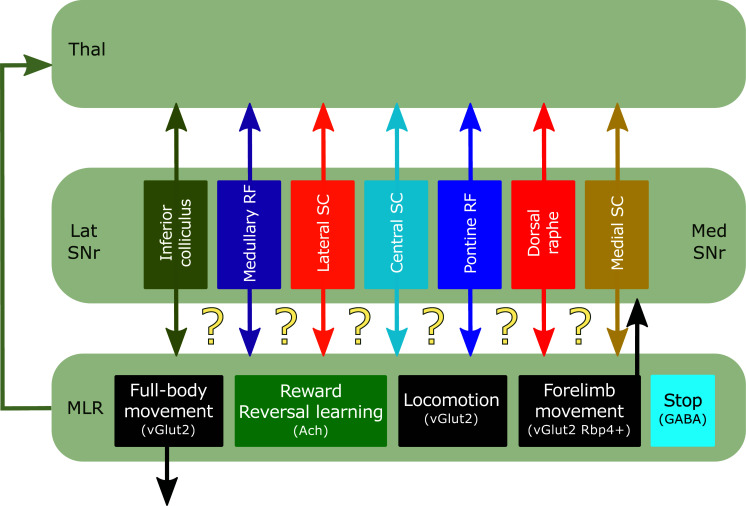
SNr projects through parallel segregated populations of inhibitory neurons to the PPN/mRT and thalamus. Axons of SNr efferent populations constitute separate channels targeting brainstem nuclei that also contact the PPN/mRT and thalamus (See [80] for more details). It remains to be determined how the independent PPN/mRT neuronal subpopulations sample these fibers and the reciprocity of the vGlut2 Rbp4+ PPN/mRT axons targeting the SNr. RF, reticular formation; SC, superior colliculus.

The SNr is the major output nucleus of the BG [80]. It integrates inputs from the direct and indirect pathways and broadcasts BG output signals. SNr outputs this information through segregated populations of neurons contacting independent brainstem structures [80]. Each of these efferent populations constitutes a dedicated channel that targets an individual brainstem nucleus while sending copies of this information to the PPN and thalamus through axonal collaterals [80]. However, the MLR neuronal subpopulation receiving these compartmentalized signals, as well as the reciprocity of those connections, is still poorly understood (Fig. **2**). In this section, we review the latest advances in our understanding of MLR and SNr interconnectivity.

#### 3.2.1 MLR Inputs to SNr

Tracing experiments have found only a sparse cholinergic PPN innervation in the SNr (Fig. **1A**), and thus far, little is known about its function [22]. On the contrary, both CnF and PPN/mRT send glutamatergic axons to SNr [19, 22, 25] (Figs. **1A** and **2**). While the CnF inputs to SNr were not thoroughly studied yet, those from PPN/mRT were dissected. Tracing experiments showed that glutamatergic neurons from the PPN and the immediately adjacent mRT send ascending projections to the SNr [25] (Fig. **1A**). Importantly, these ascending glutamatergic axons arise from an independent population of vGlut2+ neurons expressing Cre in Rbp4-Cre transgenic mice rather than bifurcating from glutamatergic neurons with descending targets (Fig. **1A** and **2**) [25]. Perturbations of ascending glutamatergic PPN/mRT neuronal activity have an entirely different effect compared to those in the descending population described above (see section 2.1.3): photo-inhibition induced immediate, uncoordinated movements of all body parts while stimulation evoked arrest of body movement regardless of the behavior that the animal was engaged in [25]. These results can be interpreted as an imbalanced modulation of BG output structures: reduction of ascending PPN/mRT excitatory drive may release the inhibition that SNr/EP impose to downstream targets leading to the execution of multiple movements simultaneously, whereas increased PPN/mRT activity may cause a general behavioral stalling by global inhibition of BG output signals. Of note, ascending Rbp4 PPN/mRT neurons are endogenously active during the execution of forelimb-associated behaviors, which contrasts with the generalized movement arrest evoked during their optogenetic activation. One reconciling explanation is that ascending Rbp4 PPN/mRT projections target specific SNr subcircuits involved in particular actions unrelated to forelimb behaviors, conveying a stop signal to competing motor commands such as locomotion [26, 80]. In this view, concurrent artificial photo-activation of multiple subcircuits may lead to generalized halting of movement [25]. Overall, these findings underscore the potential role of glutamatergic PPN/mRT in controlling BG output signals.

#### 3.2.2 SNr Inputs to MLR

SNr is the major output nucleus of the BG [80]. The SNr is located in a key position to integrate D1R- and D2R-SPNs output signals and convey this information to downstream motor targets. SNr projects through discrete populations of PV+ GABAergic neurons to multiple brainstem structures [80]. Regardless of their specific downstream synaptic target, all of these output fibers consistently send collateral branches to the diencephalon, where they universally innervate the thalamus, and the PPN/mRT [80] (Fig. **1C**), highlighting the importance of coordinating BG and MLR activity for motor control. For instance, optogenetic experiments that indirectly affected MLR neuronal activity by controlling SNr output showed that the SNr → MLR connection is a key modulator of BG-driven behavior [8]. In these experiments, unilateral photo-activation of D1R-SPNs increased the firing rate of vGlut2+ MLR neurons and induced locomotion [8]. These data suggest that the D1R-SPN inhibition of SNr inhibitory neurons projecting to MLR glutamatergic neurons has a net disinhibitory effect on vGlut2+ MLR neurons. Such disinhibition results in vGlut2+ MLR activation and movement initiation. Importantly, when MLR glutamatergic neurons were photo-inhibited during this D1R-SPN-induced locomotion, it resulted in a deceleration of the ongoing speed, probing that this neuronal population is necessary to drive BG-dependent locomotive behavior [8]. In a set of complementary experiments, bilateral photo-activation of D2R-SPNs during locomotion decreased the firing rate of vGlut2+ MLR neurons and stopped the animals throughout the illumination, indicating that D2R-SPN activation indirectly silences vGlut2+ MLR neurons. In this case, the concurrent photo-stimulation of MLR glutamatergic neurons restored locomotion, showing the sufficiency of glutamatergic MLR neurons' activation to override BG stop signals [8].

The specific synaptic neuronal targets of the SNr fibers in the MLR are not fully elucidated. Recent studies showed that vGlut2+ PPN neurons receive ipsilateral inputs from BG output structures, predominating those arising from the SNr [17, 19] (Fig. **1C** and **2**). On the other hand, vGlut2+ neurons in the CnF receive little input from the BG (Fig. **1C**) but strong input from midbrain structures in line with its proposed role in high-speed locomotion, such as escape responses [17, 80]. Less is known about the SNr inputs to cholinergic PPN neurons. Throughout the brain, the SN is the second most abundant input source to those neurons, after the colliculus [18]. However, studies investigating these inputs did not distinguish SNr from SNc, opening the possibility that part of these fibers are dopaminergic rather than GABAergic (Fig. **1C**). Finally, SNr neurons also impinge on GABAergic MLR neurons, although this connection represents a minor percentage of their complete afferent connectome [8] (Fig. **1C** and **2**).

The SNr-MLR pathway is a good example to illustrate how the combination of precise connectivity mapping and endogenous activity profiles is indispensable for understanding the information processing and computational logic of this circuit (Fig. **2**). At first glance, the reciprocal connectivity between SNr and MLR is characterized by a disinhibitory circuit, and an excitatory feedback loop could be assumed to operate as a GO circuit that conveys a start signal to downstream executed centers (Fig. **2**). In this scenario, the reduced firing of GABAergic SNr neurons releases its inhibitory control over glutamatergic PPN neurons that triggers the execution of the selected PPN-dependent movement followed by a rapid shutdown of PPN activity due to the increased drive to SNr neurons *via* their ascending collaterals. However, recordings of glutamatergic MLR neurons showed their recruitment during the execution of the chosen action [8, 17, 25]. A more parsimonious explanation can be suggested based on the existence of two discrete functionally and anatomically segregated glutamatergic PPN neuronal subpopulations in which ascending PPN collaterals target specific SNr neurons belonging to different functional subcircuits (Figs. **2** and **4**). In this alternative scenario, during the execution of forelimb-associated behaviors, disinhibition of this particular SNr channel induces the activity of ascending glutamatergic PPN neurons that may impinge on non-selected SNr subcircuits and thus silence competing motor programs, such as full-body movements. Supporting this hypothesis, subsets of SNr neurons have been found to become excited during movement execution [90, 91]. Moreover, during the performance of full-body behaviors, disinhibition of this particular SNr channel leads to the activation of descending glutamatergic PPN neurons that convey executive signals to downstream targets. Experimental support for the proposed connectivity remains to be assessed.

### 3.3 MLR - SNc Mutual Connectivity

Dopaminergic SNc neurons play a central role in goal-directed and habitual movements by modulating BG activity [92, 93]. The most studied effect of dopamine release is the one that exerts on the striatum, where it can directly positively or negatively modulate the excitability of SPNs depending on the expressed postsynaptic receptor subtype (for a review, see [94] and [76]). SNc dopaminergic inputs to the striatum also impact the synaptic release of incoming glutamatergic terminals and affect the activity of local interneurons [76, 95].

**Fig. (3):**
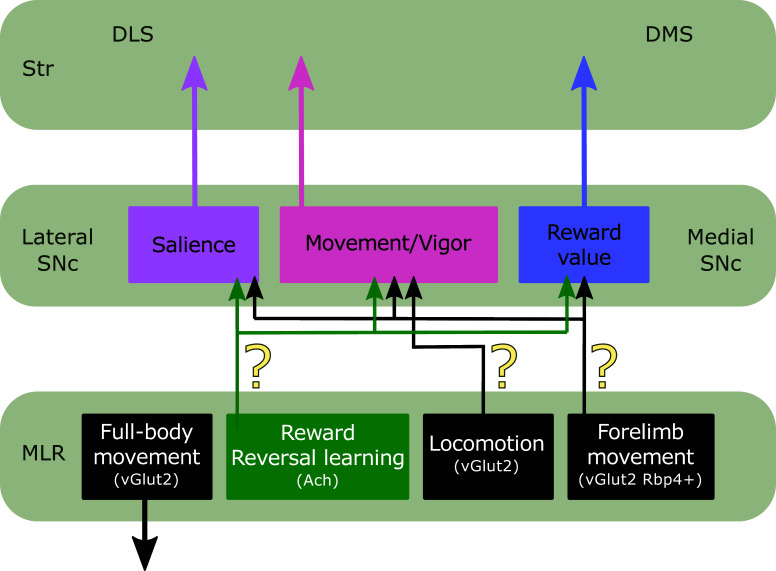
MLR-SNc-Striatum circuits are organized in parallel channels. Ascending (but not descending) projection neurons in the MLR are proposed to selectively connect to topographically organized neurons in the SNc that, in turn, contact specific striatal functional/spatial domains, conveying dedicated functions to each channel.

Importantly, the SNc is not a homogenous structure, and different functions are segregated within this region. Lateral dopaminergic SNc neurons project mainly to DLS and are recruited by both reward and aversive stimuli [96] (Fig. **3**). On the contrary, medial dopaminergic SNc neurons mostly target the DMS and present opposite responses to appetitive *versus* aversive stimuli [96] (Fig. **3**). These results are in agreement with studies performed in awake monkeys in which a shift from positive to negative responses to an aversive cue was observed when recording from lateral to medial SNc [97]. This indicates that lateral dopaminergic SNc neurons could signal the salience of an outcome regardless of its valence, while medial dopaminergic SNc neurons may encode the value of an outcome (Fig. **3**). Notably, individual dopaminergic SNc neurons are also recruited during action initiation regardless of the type of movement executed (*e.g*., locomotion, lever press), and their activity scales up with movement vigor [90, 98-100] (Fig. **3**). Importantly, dopaminergic SNc neurons responding to movement do not overlap with reward-responsive neurons [99-101], suggesting the existence of different information channels.

Previous findings have undoubtedly demonstrated that different dopaminergic neurons encode movement and reward signals which are then conveyed to the striatum to initiate movement. What is less clear is where the dopaminergic SNc neurons receive this information from (Fig. **3**). Interestingly enough, MLR neurons have been found to respond to reward, and different movement types [8, 17, 25, 68-71, 73] and could broadcast this information to BG through their direct input to SNc.

#### 3.3.1 MLR Inputs to SNc

*Ex vivo* and *in vivo* experiments demonstrated that electrical PPN stimulation induces an increase in the firing rate of SNc neurons [102-105]. What is the neuronal origin of this modulation? Several lines of evidence using conventional techniques demonstrated that SNc neurons receive direct excitatory cholinergic and glutamatergic input from PPN [103-107]. More recently, the implementation of gold-standard tracing tools allowed for a deeper understanding of this pathway. Cell-type specific monosynaptic rabies tracing studies from dopaminergic SNc neurons have identified a direct projection from PPN and mRT (Fig. **1A**) [25, 96, 108]. Both structures exhibited no bias towards DLS or DMS projecting SNc neurons [96]. The existence of a direct CnF to SNc projection has been reported in monkeys through *in vivo* antidromic stimulation experiments [69], but this connection was not confirmed in mice [96, 108]. In line with these findings, cell-type specific anterograde tracing from MLR neuronal subsets has evidenced that cholinergic and glutamatergic PPN, but not CnF neurons, project to SNc [19, 21, 22] (Fig. **1A**).

What is the physiological relevance of PPN to SNc inputs? Early experiments have used non-cell-type specific strategies to address whether encoded reward signals in PPN may flow to midbrain dopaminergic regions to impact behavior. Interestingly, it has been shown that rat PPN neurons respond to reward-related cues before dopaminergic midbrain neurons, and silencing PPN activity suppresses dopaminergic response to cues [109]. Moreover, neuronal activity recordings in monkeys reported SNc-projecting PPN neurons that encode reward and sensorimotor signals [69]. These findings support a pathway in which reward-related sensory information is transmitted from PPN to SNc.

Cholinergic PPN neurons convey direct excitatory input mediated by nicotinic receptors to both the dorsal and ventral tiers of the SNc in rats [21] (Fig. **1A**). Also, acetylcholine released through PPN terminals was shown to elicit an indirect excitatory effect on glutamatergic terminals in SNc [21]. The functional relevance of this pathway was assessed by optogenetic stimulation or inhibition of cholinergic PPN axonal terminals onto ventral SNc (vSNc), which evokes an increase or a reduction of locomotion in the open field, respectively [21]. Notably, photo-excitation of cholinergic PPN-to-vSNc terminals has no impact on a conditioning place preference task, whereas stimulation of PPN-to-VTA terminals reduces place aversion [21]. These findings are in line with the known connectivity of vSNc that preferentially targets DLS [110] (Fig. **3**). In summary, cholinergic PPN-to-vSNc input positively modulates locomotion, and cholinergic PPN-to-VTA projection mediates reward reinforcement [21]. This study is a good example of how the multifaceted roles of cholinergic PPN neurons can be parsed out by selective targeting of circuit elements.

PPN cholinergic inputs to SNc also contribute to cognitive flexibility [73]. Optogenetic terminal stimulation of PPN cholinergic afferents in the SNc significantly improves reversal learning [73] (Fig. **3**). In contrast to Xiao’s findings, the photo-activation protocol used had no effect on locomotion, which could be related to differences in the specific SNc tier stimulated. Altogether, these findings indicate a dual role of PPN cholinergic input to SNc. While projections to vSNc affect movement initiation and vigor, other projections targeting different tiers may carry reinforcement signals (Fig. **3**).

Cholinergic PPN neurons are thought to be a homogeneous population broadcasting signals to a wide range of targets. Contrarily, it was also shown the existence of non-overlapping populations projecting to SNc (see above), and VTA [21] opening the possibility that cholinergic subpopulations could be divided by their synaptic targets rather than for their expression of markers (Fig. **3**). An interesting open question is whether branches from the same cholinergic axons innervate the striatum and the SNc. In that case, convergent signals could reach the striatum directly through the cholinergic connection to the striatum and indirectly through the cholinergic PPN → SNc → Striatum loop. If this is true, both acetylcholine and dopamine release could be spatial/temporally coordinated on synergic or antagonistic subnetworks of striatal neurons, favoring different computations upon PPN activation.

Glutamatergic PPN neurons are also able to significantly control the spiking activity of dopaminergic SNc neurons. For example, early studies have shown that the increased firing of SNc neurons upon PPN electrical stimulation is blocked by ionotropic glutamate receptor antagonists [105, 111]. More recently, it has been shown that optogenetic stimulation of glutamatergic PPN afferents to dopaminergic SNc neurons can evoke burst spiking activity [112] (Fig. **1A**). Finally, acetylcholine released from PPN terminals may act presynaptically to regulate incoming glutamatergic fibers from PPN or other sources [21] (Fig. **1A**).

Dopaminergic SNc neurons also receive direct inhibition from GABAergic PPN neurons, resulting in reduced dopamine release in the caudal dorsal striatum [45] (Fig. **1A**). Behaviorally, photo-activation of GABAergic PPN terminals on SNc reduced exploratory locomotion in the open field without affecting other behaviors [45]. Notably, stimulation of SNc projecting GABAergic PPN terminals prevents the initiation and execution of a learned lever press sequence [45]. These results are interpreted by the authors as an inhibition to execute purposive behaviors likely due to a change in action value [45].

In summary, dopaminergic SNc neurons receive signals from GABAergic, cholinergic, and glutamatergic PPN neurons that impact movement, reinforcement learning, and cognitive flexibility [21, 45, 73]. Thus, it is conceivable that dopaminergic SNc neurons integrate sensory, reward, and movement information encoded by cholinergic and glutamatergic PPN neurons. But what exact type of information is transmitted to SNc from PPN? It was found in monkeys that SNc projecting PPN neurons respond to visual, auditory, and tactile stimuli, although the neurotransmitter phenotype of these neurons was not assessed [69]. Moreover, cholinergic PPN neurons not only encode reward information but also changes stimulus-outcome contingency [73]. Furthermore, glutamatergic PPN neurons are recruited during locomotion and other self-initiated actions such as rearing, grooming, and handling [17, 25]. However, due to the presence of two segregated glutamatergic subpopulations with ascending or descending projections, not all this information reaches the SNc [25]. The SNc may receive direct forelimb-associated movement signals from SN-projecting Rbp4 PPN/mRT neurons. On the contrary, rearing and explorative locomotion information encoded by spinally projecting glutamatergic PPN/mRT neurons have no direct access to SNc [17, 25]. Moreover, information from fast locomotor events related to escape responses encoded by glutamatergic CnF neurons does not give feedback, at least directly, to SNc [17, 19]. Altogether, the functional heterogeneity of PPN neuronal subpopulations is also reflected in the diversity of signals transmitted to dopaminergic SNc neurons. Whether this diverse but precise information influences SNc activity globally or whether the information flow is organized in subcircuits involved in specific behaviors remains to be determined.

#### 3.3.2 SNc Inputs to MLR

The first description of dopaminergic SNc input to the MLR was performed by Rolland and collaborators, who found dopamine transporter-positive SNc fibers in the PPN and CnF of monkey brains [113]. This pathway is highly relevant because it may imply that PPN dysfunction in Parkinson's disease could arise not only from impaired BG output function but also from the direct loss of dopaminergic modulatory innervation. This connection has been recently confirmed in mice and rats. Monosynaptic rabies tracing from specific MLR neuronal subsets has revealed the existence of a direct SNc projection to glutamatergic and cholinergic PPN neurons in addition to glutamatergic CnF neurons [17-19] (Fig. **1C**). Moreover, retrograde viral tracing from PPN has shown TH+ labeled neurons in SNc [114], indicating their dopaminergic phenotype. Interestingly, double retrograde labeling from the striatum and PPN using two traditional retrograde tracers demonstrated that most SNc neurons send bifurcating axons targeting not only the BG but also the PPN in rats [114]. From a functional perspective, stimulation of SNc elicited dopamine release in the rat PPN [114]. Moreover, in lampreys, dopamine release from upstream circuits acts on D1 receptors to increase the locomotor output [13]. However, whether all identified pathways from SNc to specific MLR neuronal subpopulations are dopaminergic and the receptor type involved in these connections remains to be determined [17-19].

### 3.4 MLR Connectivity with Other BG Nuclei

#### 3.4.1 Weak MLR Inputs from STN, EP, and GPe

Rabies tracing experiments from specific neuronal subpopulations have demonstrated that there is an overall weak projection from STN, EP, and GPe to MLR neurons compared to other BG nuclei [8, 17-19]. Of these, the most notable one is the STN projection to glutamatergic and cholinergic PPN neurons [17-19]. The functional relevance of this sparse input remains to be determined.

#### 3.4.2 MLR Projections to STN and EP

In contrast to the scarce input received from STN and EP, specific MLR neurons have a robust projection to these structures. Early experiments based on traditional techniques have shown that GABAergic, cholinergic, and glutamatergic PPN/mRT neurons convey axonal projections to STN [54, 115, 116]. Furthermore, MLR projections to EP have been located in the mRT but not in the PPN subregion [29]. More recently, modern anterograde tracing experiments from specific neuronal subpopulations have demonstrated that glutamatergic PPN/mRT but not glutamatergic CnF neurons, heavily project to EP and STN [19, 22], in line with the observed connectivity bias with other BG structures. Notably, SN-projecting Rbp4+ glutamatergic PPN/mRT neurons send prominent collaterals to STN and EP [25] (Fig. **1A**). Moreover, a previous study showed that neurochemically distinct subpopulations of PPN neurons convey differential innervation to either STN or the medullary gigantocellular nucleus [54]. These findings demonstrate that divergent information is transmitted to upstream or downstream targets: while forelimb-related information is broadcasted simultaneously to SN, STN, and EP, downstream structures receive segregated signals related to full-body movements. What is the net effect of ascending STN projections? *In vitro* experiments have shown that PPN stimulation induces an overall excitatory response in STN neurons [117]. Therefore, PPN input to STN neurons may increase the drive of the indirect pathway leading to increased inhibition of BG output targets. A similar effect is achieved by the efferent projection to SNr and EP [25].

## CONCLUSION AND OUTLOOK

In this review, we have described recent studies using cell type-specific strategies to unravel the endogenous role of MLR. In doing so, we uncovered a high degree of heterogeneity in the input-output network and function of the individual circuit components encompassing the MLR. Although the initial finding of a locomotion-inducing site justified the coined term “Mesencephalic Locomotor Region,” current advances in the field support a new conceptual framework in which divergent functions are encoded by precise circuit elements embedded within the MLR. On the one hand, glutamatergic CnF neurons control escape behavior triggered by fast-incoming sensory information and/or defense circuit signals, most likely *via* descending medullary targets. On the other hand, descending glutamatergic PPN and mRT neurons contribute to postural adjustments and slow exploratory behaviors but lack the capability of initiating motor output on their own, whereas ascending glutamatergic PPN and mRT neurons convey feedback to BG on forelimb movements. Moreover, cholinergic PPN neurons contribute to reinforcement learning, reward, and locomotion. Finally, GABAergic PPN neurons may modulate behavioral output through local inhibition of movement-related neurons or by its direct action on SNc. Whether these microcircuits work as parallel channels of behavioral output or operate in concert to orchestrate adaptive motor responses, remains to be determined. Until then, we suggest using the term MLR with caution since it may lead to the inappropriate understanding of a single homogeneous center with a unique function.

We continued by describing the strong interplay existing between BG and MLR structures. In this process, we have underscored the high degree of heterogeneity and specificity in the subcircuits involved in this interaction (Fig. **1**). Of note, two MLR neuronal subsets fall outside this description: the glutamatergic CnF and the GABAergic neurons. There is consensus evidence demonstrating that glutamatergic CnF neurons controlling escape responses are mainly driven by midbrain inputs and display rare connections with BG [17, 19, 25]. Moreover, GABAergic MLR/PPN neurons only receive from BG a weak SNr input and convey selective feedback to SNc [8, 45]. At the other end of the spectrum, glutamatergic and cholinergic PPN/mRT neurons establish ample reciprocal connections with most BG nuclei (Fig. **1**). The robust anatomical and functional data summarized in this review strongly antagonize the generalized concept that the MLR functions just like an output structure of BG. While some subcircuits transmit BG motor commands to downstream centers eliciting movement, such as descending glutamatergic PPN neurons, the ascending glutamatergic, cholinergic, and GABAergic pathways have a strong modulatory effect on BG function.

The emerging principle of motor system organization posits that specific subcircuits within the BG network encode and control particular movements [27, 78]. These action-specific pathways are sometimes organized topographically [26]. However, they are also likely to be embedded within intermingled neuronal subpopulations making them more difficult to disentangle. In this model, movement-specific BG subcircuits transmit the corresponding output signal to the appropriate downstream brainstem structure to elicit a particular behavior. In turn, these downstream motor centers act as parallel command lines controlling specific movement elements, linking upstream centers with spinal executive circuits [27]. Several examples of brainstem command lines controlling specific behaviors have been recently described, including reaching, grasping, turning, orienting, high-speed locomotion, behavioral arrest, and urine release [43, 118-123]. In this context, the PPN may serve as one of the command lines controlling explorative locomotion, rearing, and postural adjustment. Still, as discussed above, the sophisticated wiring diagram between BG and MLR argues in favor of a more complex framework in which particular components of PPN/mRT are linked to precise BG action channels and therefore contribute not only to the top-down execution of motor programs but also to the bottom-up modulation of BG function. A key example of this concept can be found in the glutamatergic PPN/mRT projection to SNr. In this pathway, the activity of ascending glutamatergic PPN/mRT neurons drives the excitation of GABAergic SNr neurons that is traditionally assumed to lead to a generalized synchronic increased inhibition of downstream targets. However, the SNr is a highly organized structure [80]. Therefore, it is tempting to speculate that ascending glutamatergic PPN/ mRT neurons may target specific SNr domains impacting the behavioral output in an action-selective manner [80]. Given that ascending glutamatergic PPN/mRT neurons are endogenously recruited during the execution of forelimb movements, it is not logical to think that they may inhibit their own subcircuit since this would lead to the halting of the current action. Instead, it is conceivable that they selectively impinge and, thus, inhibit SNr output channels involved in different (competing) behavioral repertoires. In this way, ascending glutamatergic PPN/mRT neurons may contribute to the role of BG in action selection.

It has long been postulated that motor centers establish axon collaterals that ascend the neuraxis conveying an “efference copy” (also named “corollary discharge”) of the to-be-performed action at multiple levels [124]. An efference copy of the planned movement serves two purposes: to inform the sensory system of the upcoming self-generated environment displacement such that it can perform the appropriate adjustments to render a stable perception of the world and to update higher-order executive motor centers of the imminent movement in order to plan and calibrate the subsequent command during action sequences [124]. Thus, in both cases, a corollary discharge must carry an accurate copy of the motor command information transmitted to the output executive circuits. Ascending MLR projections were hypothesized to carry such a copy of the locomotor parameters. Indeed, it has recently been shown that locomotion speed information from PPN is conveyed to the navigational system of the entorhinal cortex *via* the basal forebrain, which might be relevant for path integration [47]. Moreover, subthreshold optogenetic stimulation of glutamatergic MLR neurons was sufficient to elicit an increase in visual responses in the primary visual cortex through its basal forebrain projections, which may underlie a shift in attention during sensory processing [42]. Therefore, MLR ascending collaterals may potentially provide an efference locomotor copy to couple the cortical state with the motor output. However, this viewpoint contrasts with recent findings demonstrating that glutamatergic MLR neurons promoting locomotion and body stretching convey these signals to the medullary reticular formation and spinal cord but not to the SN [17, 25]. Indeed, the activity of ascending glutamatergic PPN/mRT neurons projecting to SN is selectively tuned to forelimb-associated movements [25]. Hence, so far, there is no evidence for a direct MLR efference copy of locomotor signals to higher-order motor centers such as the BG. Instead, SN-ascending glutamatergic PPN/mRT neurons may serve a role other than simply broadcasting performed locomotor movements, such as contributing to action selection, as discussed above.

Another important point to consider is that the neuronal heterogeneity described so far within the PPN/mRT may just reflect the tip of the iceberg. There is evidence indicating that different ascending glutamatergic neuronal subpopulations may coexist within this region. Thalamus-projecting glutamatergic PPN/mRT neurons were shown to encode cue signals [74]. Glutamatergic MLR neurons projecting to the basal forebrain were found to have a strong impact on visual cortex processing [42] and to convey locomotion speed signals to the entorhinal cortex [47]. Striatally-projecting glutamatergic PPN neurons decreased SPN firing through a disynaptic pathway inhibiting striatal output [23]. Moreover, Rbp4+ glutamatergic PPN/mRT neurons modulate the drive of SN, which leads to a strong influence of overall behavioral output [25]. Nevertheless, it remains to be determined the extent of overlap between these a priori different neuronal subpopulations. The combination of cell-type and projection-specific tools to target precise neuronal subpopulations may help understand general rules governing the mechanisms underlying sensory-motor integration.

**Fig. (4):**
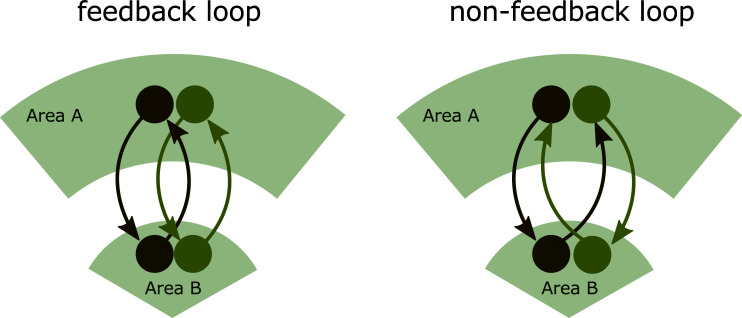
Interconnection between areas does not imply reciprocal microcircuit connectivity. (**A**) Schematics of two distant interconnected areas where the neurons are reciprocally connected (feedback loop). (**B**) Schematics of two distant areas where the neurons are not reciprocally connected (non-feedback loop).

A largely overlooked issue when considering the reciprocity of BG-PPN/mRT connectivity is whether or not feedback loops exist in particular pathways or subcircuits (Fig. **4**). As exemplified in the figure, neurons residing in interconnected structures do not necessarily convey feedback to the same microcircuit. Given the complexity and unveiling heterogeneity of BG and MLR circuits, solving this dichotomy may require precise identification of the circuit elements involved in both interconnected structures (Fig. **4**). Throughout the review, we have pointed to the missing pieces of available anatomical studies to fulfill this knowledge gap. Fortunately, current technological advances allow an unprecedented level of cellular resolution by combining topographical organization, projection specificity, and molecularly defined neuronal subpopulations to uncover the anatomical heterogeneity of neural ensembles and disentangle intricate input-output connectivity maps. It is also relevant to assess the functional impact of identified connections on circuit activity, which may uncover modulation of firing properties [112], network dynamics, or presynaptic effects on neurotransmitter release [21]. This knowledge will be instrumental in helping understand the specific computation of each precise subcircuit and predict how this information is integrated with other circuit players to produce an adaptive behavioral output, paving the way to uncover the organizing principle of the motor system and its deficits during disease.

## LIST OF ABBREVIATIONS

Ach: Cholinergic Neuron
ARAS: Ascending Reticular Activating System
BG: Basal Ganglia
ChIs: Cholinergic Interneurons
CnF: Cuneiform Nucleus
D1R: Dopamine-1-receptor
D2R: Dopamine-2-receptor
DLS: Dorsolateral Striatum
DMS: Dorsomedial Striatum
EP: Entopeduncular
FSI: Fast Spiking Interneuron
GPe: Globus Pallidus Externus
LTS: Low-threshold Spike Interneuron
MLR: Mesencephalic Locomotor Region
mRT: Mesencephalic Reticular Formation
NAc: Nucleus Accumbens
PPN: Pedunculopontine Nucleus
PV: Parvalbumin Neuron
REM: Rapid Eye Movement
SNc: Substantia Nigra Compacta
SNr: Substantia Nigra Reticulate
SPN: Spiny Projection Neuron
STN: Subthalamic Nucleus
TH: Tyrosine Hydroxylase
vGlut2: Glutamatergic
